# Are Maternal Thyroid Autoantibodies Generated by PCBs the Missing Link to Impaired Development of the Brain?

**DOI:** 10.1289/ehp.112-a862a

**Published:** 2004-11

**Authors:** Janna G. Koppe

**Affiliations:** ECOBABY Society, Amsterdam, the Netherlands, E-mail: janna.koppe@inter.nl.net

In her interesting review addressing endocrine disruption and the developing brain, Colborn (2004) asked rightly for special attention to the role of a disruption of thyroid hormones and thyroid hormone metabolism, which negatively influence early development of the fetal brain. As mechanisms of action, chemicals such as polychlorinated biphenyls (PCBs) were discussed in a dose-related way; the higher the exposure level of the mother, the more problems of brain development will be found in the baby (Colborn 2004). However, this is not always true. Patandin et al. (1999) found a four-point decline in IQ at 4 years of age in relation to maternal PCB levels in the Netherlands. In a follow-up study of Faroese children at 7 years of age, Grandjean et al. (1997) found no relation of PCBs with cognitive impairment; the levels of PCBs were almost 4 times higher in the Faroese population than in the Dutch population (Longnecker et al. 2003).

One explanation of the missing link might be that effects of PCBs are not directly toxic but instead are toxic through immunomodulatory mechanisms in the mother. In a comment on the impact of maternal PCB and dioxin exposure on the neonate’s thyroid hormone status, Vulsma (2000) noted that PCBs affect the generation of autoantibodies against thyroid tissue [e.g., thyroid peroxidase antibodies (TPO-Ab)]. In a study in Slovakia, Langer et al. (1998) described an increase in TPO-Ab in relation to PCB exposure. These antibodies do pass through the placenta.

An important risk factor for impaired infant development is a low free thyroxine (fT_4_) concentration in early pregnancy; particularly at risk are the mothers with low fT_4_ and high TPO-Ab titers. These anti-bodies are found in 10% of (euthyroid) women at 12 weeks’ gestation in the Netherlands (Pop et al. 1995, 1999). To my knowledge, none of the studies on effects of PCBs in human pregnancy have reported data on maternal TPO-Ab titers.

If the findings reported by Colborn (2004) can be explained by autoimmune processes that cause low fT_4_ in the mother and negatively affect her developing baby, then it seems more logical that prenatal PCB exposure is related to developmental impairment instead of the amount of PCBs transferred by breast milk after birth.

I agree with Colborn (2004) that all women who plan to become pregnant should be evaluated for thyroid hormone status.
